# Aging in Place for Older Subsidized Housing Residents: The Influence of Social Connection and Social Environment

**DOI:** 10.1093/geroni/igab046.522

**Published:** 2021-12-17

**Authors:** Rebecca Brown, David Reyes-Farias, Erin Finucane, Amanda Watson, Momana Jahan, Sonia Pandit, Carolina Reid, Barbara Resnick

**Affiliations:** 1 Perelman School of Medicine of the University of Pennsylvania, Wynnewood, Pennsylvania, United States; 2 University of Pennsylvania, Philadelphia, Pennsylvania, United States; 3 University of California, Berkeley, Berkeley, California, United States; 4 University of Maryland School of Nursing, Baltimore, Maryland, United States

## Abstract

Older adults living in subsidized housing experience health disparities including disproportionate rates of social isolation and nursing home admission. Little is known about how social relationships and social environment influence aging in place for this population. We interviewed 58 residents aged 62 or older. Qualitative thematic analyses revealed that social relationships both inside and outside the building contributed to residents’ experience of aging in place. Relationships with other residents and staff members provided social support, while connections to family and friends outside the building “opened up” the residents’ world and provided a sense of connection to the larger community. Social and physical environment also contributed, with residents’ ability to move between private and public spaces leading to feelings of freedom and independence. Discussion focuses on expanding definitions of aging in place to encompass residents’ experiences and implications for improving aging in place for this population.

